# Association between Socioeconomic Status and 30-Day and One-Year All-Cause Mortality after Surgery in South Korea

**DOI:** 10.3390/jcm7030052

**Published:** 2018-03-10

**Authors:** Tak Kyu Oh, Kooknam Kim, Sang-Hwan Do, Jung-Won Hwang, Young-Tae Jeon

**Affiliations:** Department of Anesthesiology and Pain Medicine, Seoul National University Bundang Hospital, 166, Gumi-ro, Bundang-gu, Seongnam 463-707, Korea; airohtak@hotmail.com (T.K.O.); 54271@snubh.org (K.K.); shdo@snu.ac.kr (S.-H.D.); jungwon@snubh.org (J.-W.H.)

**Keywords:** mortality, marriage, general surgery

## Abstract

Preoperative socioeconomic status (SES) is associated with outcomes after surgery, although the effect on mortality may vary according to region. This retrospective study evaluated patients who underwent elective surgery at a tertiary hospital from 2011 to 2015 in South Korea. Preoperative SES factors (education, religion, marital status, and occupation) were evaluated for their association with 30-day and one-year all-cause mortality. The final analysis included 80,969 patients who were ≥30 years old, with 30-day mortality detected in 339 cases (0.4%) and one-year mortality detected in 2687 cases (3.3%). As compared to never-married patients, those who were married or cohabitating (odds ratio (OR): 0.678, 95% confidence interval (CI): 0.462–0.995) and those divorced or separated (OR: 0.573, 95% CI: 0.359–0.917) had a lower risk of 30-day mortality after surgery. Similarly, the risk of one-year mortality after surgery was lower among married or cohabitating patients (OR: 0.857, 95% CI: 0.746–0.983) than it was for those who had never married. Moreover, as compared to nonreligious patients, Protestant patients had a decreased risk of 30-day mortality after surgery (OR: 0.642, 95% CI: 0.476–0.866). The present study revealed that marital status and religious affiliation are associated with risk of 30-day and one-year all-cause mortality after surgery.

## 1. Introduction

Socioeconomic status (SES) encompasses all factors that can affect an individual’s social and economic status, such as education, income, and occupational factors [1]. Thus, SES is thought to be closely related to health outcomes [2]. For example, socioeconomic inequality can affect mortality and morbidity, which highlights SES as an important global public health issue [3,4,5]. 

Postoperative outcomes are affected by various factors, with physiological status being one of the most important [6]. Although many studies have examined the effects of physiological factors, few studies have examined the relationship between SES and outcomes after surgery. For example, some studies have suggested that outcomes after pediatric surgery [7] or hip joint arthroplasty [8] are influenced by the patients’ primary payer status. Other studies have revealed that a low SES is associated with poor cancer-specific survival among patients with colorectal cancer [9]. In addition, a more recent population-based cohort study suggested that frailty and poverty are associated with higher risks of one-year mortality after major elective noncardiac surgery [10]. Furthermore, lower SES is reportedly correlated with poor outcomes after surgery [11,12]. However, the previous studies have focused on this relationship in terms of economic and/or payer status [8,9,10,11,12], and it is important to be aware that this relationship may exhibit regional or national differences. 

Korea has a national health insurance system that provides patients access to treatment (including surgery), regardless of disease severity [13]. Furthermore, Korean people are known to have relatively high educational levels [14]. Therefore, it is possible that, in the Korean population, there may be a unique relationship between SES and mortality after surgery that has not been considered in previous studies. Therefore, the present study examined the associations between SES characteristics and both 30-day and one-year all-cause mortality after surgery among adults who were treated at a Korean tertiary hospital.

## 2. Materials and Methods

This study’s retrospective protocol was approved by the institutional review board of the Seoul National University Bundang Hospital (SNUBH; B-1705/395-106) and adhered to the tenets of the Declaration of Helsinki. Since 2003, SNUBH has maintained an electronic medical record system, and approximately 150 surgeries are performed each day in the 38 operating rooms. Data were obtained from the medical records of patients ≥30 years old who had undergone elective surgical treatment at SNUBH between 1 January 2011 and 31 December 2015. Only the last surgery was considered for patients who underwent surgery more than once during the study period. Patients were excluded if their medical records were incomplete or if they did not agree to the collection of their information at the time of admission.

## 3. Data Collection and Outcomes

Data were collected regarding the patients’ demographic characteristics (sex, age, body mass index) and clinical characteristics (Charlson comorbidity index, American Society of Anesthesiologists classification, surgery type, preoperative estimated glomerular filtration rate, anesthesia type, postoperative intensive care unit admission, date of death). These data were extracted from the surgical or preanesthetic registries. In addition, patients were requested to complete an SES-related interview at their admission, although each patient had the right to refuse the release of their SES data. All data were recorded by trained nurses and maintained in the nursing records. The present study examined data regarding educational levels (less than high school, high school to less than college, college or higher), occupation (office worker, professional job [licensed job], housework, self-employed, student or military, unemployed), marital status (never married, cohabitating or married, divorced or separated, widowed), religion (Protestant, Catholic, Buddhism, others (Hinduism, Islam, Cheondogyo, none), alcohol consumption (yes, no, quit), and smoking habit (yes, never smoked, quit).

The data collection was performed by medical record technicians who were blinded to the study’s purpose. The exact dates of death for patients were obtained with the approval of the Korean Ministry of the Interior and Safety. The primary outcomes for the present study were defined as the associations between preoperative SES factors and both 30-day and one-year all-cause mortality after surgery. 

## 4. Statistical Methods

All data were reported as number (percent) or mean ± standard deviation. Univariate logistic regression analyses were used to identify relationships between the patients’ characteristics and 30-day or one-year mortality after surgery. Multivariable logistic regression analyses were then performed using the significant variables (*P* < 0.1) from the univariate analyses. All analyses were performed using IBM SPSS software (version 23.0; IBM Corp., Armonk, NY, USA), and differences were considered statistically significant at a *P*-value of < 0.05.

## 5. Results

The present study evaluated data from 86,735 patients who were ≥30 years old at their SNUBH admission, and a total of 108,260 surgical cases were identified. When any patient underwent two or more surgeries, only the medical record for the final surgery was included. In addition, 5766 cases were excluded because of inaccurate preoperative SES information and personal information protection. Thus, 80,969 patients were included in the final analysis, and their baseline characteristics are presented in Table 1. A total of 339 patients (0.4%) died within 30 days after surgery, and 2687 patients (3.3%) died within one year after surgery. 

## 6. 30-Day and One-Year Mortality after Surgery

The simple relationships between the patients’ characteristics and 30-day mortality after surgery were analyzed using univariate logistic regression analyses (Appendix A), and significant characteristics were subsequently included in the multivariate logistic regression analyses (Table 2). Age was significantly associated with 30-day mortality after surgery (odds ratio (OR): 1.012, 95% confidence interval (CI): 1.003–1.021, *P* = 0.006). In addition, as compared to nonreligious patients, Protestant patients had a significantly lower risk of 30-day mortality after surgery (OR: 0.642, 95% CI: 0.476–0.866, *P* = 0.004). As compared to never married patients, those who were married or cohabitating (OR: 0.678, 95% CI: 0.462–0.995, *P* = 0.047) and those divorced or separated (OR: 0.573, 95% CI: 0.359–0.917, *P* = 0.02) had a lower risk of 30-day mortality after surgery. Moreover, patients who consumed alcohol had an increased risk of 30-day mortality after surgery (OR: 1.390, 95% CI: 1.048–1.844, *P* = 0.022) than did patients who did not. 

The simple relationships between the patients’ characteristics and one-year mortality after surgery were analyzed using univariate logistic regression analyses (Appendix B), and Table 3 shows the results of the multivariate logistic regression analyses for one-year mortality after surgery. Among the SES characteristics, only being married or cohabitating was associated with a decreased risk of one-year mortality after surgery (OR: 0.857, 95% CI: 0.746–0.983, *P* = 0.028). The Hosmer-Lemeshow test results were appropriate (*P* > 0.05). 

## 7. Discussion

The present study demonstrated that several SES characteristics were significantly associated with 30-day and one-year all-cause mortality after surgery. For example, reduced risks of 30-day mortality after surgery were observed among patients who were Protestants (vs. nonreligious), married or cohabitating (vs. never married), divorced or separated (vs. never married), and those who did not consume alcohol (vs. patients who did). However, a reduced risk of one-year mortality after surgery was only observed among patients who were married or cohabitating (vs. never married patients). None of the other variables, such as educational level or occupation, were associated with 30-day or one-year all-cause mortality. Our study is valuable, in that all Korean patients are covered by the national health insurance system, regardless of their income. Therefore, there was equal access for Korean patients to tertiary care hospitals in receiving surgical procedures regardless of the preoperative SES factors.

Marital status has been reported to improve mortality after surgery in cases that involve radical cystectomy [15] or elective colectomy [16], but not in cases that involve esophagectomy [17]. However, those studies are limited by their focus on specific diseases. In contrast, the present study only included adult patients (≥30 years old) who underwent surgery at a tertiary hospital during a five-year period, which provides a broader evaluation of the relationship between SES and mortality after surgery. The present study revealed that never-married patients were at increased risk of 30-day and one-year all-cause mortality after surgery. Interestingly, a recent study evaluated the association between marital status and mortality after noncardiac surgery among 11,588 American patients and revealed a 31% higher risk of mortality among male unmarried patients [18]. In contrast, we did not detect a significant difference in mortality risk when we compared men and women according to their marital status. 

The improved outcomes among married patients may be attributed to the psychological, economic, and social benefits of marriage [19,20]. In addition, given the relationship between marital status and 30-day mortality (which is similar to in-hospital mortality), social and psychological support appear to affect both everyday situations and needs during hospital stays. Furthermore, emotional support is a strong independent predictor of prognosis among elderly patients who are hospitalized because of heart failure [21]. Thus, the positive effects of being married on 30-day mortality are understandable. Nevertheless, it is intriguing that the same benefit was observed among divorced or separated patients (vs. the never-married group), and further studies are needed to examine this issue, as divorce is generally thought to be related to negative health outcomes [22]. It is possible that divorced or separated patients receive emotional and psychological support from their children, which might be related to better support and less stress, whereas never-married patients may not receive equivalent support. 

The present study revealed that Protestant patients had a decreased risk of 30-day mortality after surgery, as compared to nonreligious patients, which may be related to emotional support. In this context, Protestantism is the most popular and vibrant religion in Korea [23], and the weekly worship services at SNUBH are attended by a large number of patients and caregivers. A previous meta-analysis also suggested that religious involvement reduces mortality [24], which supports our findings that Protestantism was associated with a decreased risk of 30-day mortality after surgery. 

Another interesting finding was that current alcohol use, but not smoking status, influenced the risk of mortality after surgery. In Korea, alcohol consumption is associated with increased risks of mortality, including cancer-related mortality [25]. Although smoking is also a known risk factor for mortality [26], its effects are not typically observed during relatively short follow-up periods (e.g., the 30-day and one-year periods that we studied). Furthermore, it is recommended that patients who undergo surgery with anesthesia at SNUBH stop smoking, given the risks that are associated with smoking in this setting [27]. Thus, this policy might have eliminated any effect of smoking on 30-day mortality. 

Lastly, an interesting fact concerning our study is that, preoperative American Society of Anesthesiologists (ASA) classification and Charlson comorbidity index scores were not associated with 30-day and 1-year mortality after surgery. ASA classification and Charlson-related comorbidity are known important risk factors of mortality after surgery [28,29]. To some extent, it might be explained that, our study included all patients, regardless of the duration or risk of operation among non-cardiovascular surgeries. In general, previous studies were performed for specific surgeries or populations to explain the impact of the preoperative comorbidities of patients [28,29,30]. Our study aimed to know the impact of preoperative SES factors on the outcome after surgery, thus, the inclusion of the general population might have reduced the effect of the physical comorbidity in our study. However, further study is needed to show the effect of preoperative SES factor in addition to preoperative comorbidities.

The present study has several limitations. First, the retrospective design is associated with a known risk of bias. Second, data were only obtained from a single tertiary hospital in South Korea, and the results may not be generalized to broader and more diverse patient populations in other countries. Third, we were unable to obtain accurate causes of death for all patients; therefore, we could only analyze all-cause 30 day and one-year mortality, rather than disease-specific mortality. Furthermore, it is possible that all-cause one-year mortality after surgery is less likely to be due to the procedure itself and much more likely to be due to age and co-existing disease. Fourth, income data of patients were not collected and could not be included in the analyses. Lastly, our study did not analyze specific procedure-related and comorbidity-related variables such as the length of surgical procedures, risk classification, estimated blood loss, and serious cardiac and pulmonary comorbidities. Therefore, it is possible that the significant findings may be due to unknown associations with other unaccounted for variables. Nevertheless, the present study provides valuable information regarding the associations between SES characteristics and both 30-day and one-year mortality after surgery among Korean patients who underwent both cardiac and noncardiac surgery.

## 8. Conclusions

In conclusion, we found that the never-married status was associated with increased risks of 30-day and one-year all-cause mortality after surgery among Korean patients ≥30 years old, as compared to patients who were married or cohabitating. In addition, as compared to nonreligious patients, Protestant patients had a decreased risk of 30-day all-cause mortality after surgery. 

## Figures and Tables

**Table 1:** Baseline characteristics of the total study population.

Variables		*N* or Value	Percent or SD
Gender	Male/Female	35,281/45,688	43.6%/56.4%
Body mass index (kg/m^2^)		24.06	3.55
Age (year)		54.44	16.23
Charlson comorbidity index score		0.57	1.085
ASA Class	I	36,959	45.6%
	II	39,330	48.6%
	≥III	4680	5.8%
Type of Surgery	Cardiovascular surgery	1138	1.4%
	General surgery	19,474	24.3%
	Neurosurgery	2847	3.6%
	Spine surgery	4329	5.4%
	Thoracic surgery	3913	4.9%
	Procedures *	601	0.8%
	Orthopedic surgery	12,346	15.4%
	ENT, Plastic, Dental, OPH	19,514	24.4%
	Urology or OBGY	15,839	19.8%
Preoperative eGFR (mL/min/1.73 m^2^)	>90	50,249	62.1%
	60–90	25,628	31.7%
	30–60	3597	4.4%
	<30 or RRT	1495	1.8%
Type of Anesthesia	General anesthesia	54,879	67.8%
	Regional anesthesia	10,644	13.1%
	Monitored anesthesia care	15,446	19.1%
Postoperative ICU admission	Yes	2982	3.7%
	No	77,987	96.3%
Education Level	Less than high school	21,191	26.2%
	More than or equal to high school, less than college	32,400	40.0%
	More than, equal to college	27,378	33.8%
Occupation at Surgery	Office worker	14,874	18.4%
	Professional	4284	5.3%
	Housework	25,582	31.6%
	Own business	15,905	19.6%
	Student or military	2567	3.2%
	Unemployed	17,757	21.9%
Marital Status	Never married	15,049	18.6%
	Married, living together	55,558	68.6%
	Divorced/separated	6459	8.0%
	Widowed	3903	4.8%
Religion	Yes	36,017	44.5%
	No	44,952	55.5%
Classification of Religion	Protestantism	16,253	20.1%
	Catholicism	7522	9.3%
	Buddhism	11,962	14.8%
	Others **	280	0.3%
	None	44,952	55.5%
Current Alcohol Use	Yes	21,277	26.3%
	No	39,138	48.3%
	Quit	20,554	25.4%
Smoking History	Yes	17,647	21.8%
	Never smoked	42,856	52.9%
	Quit	20,466	25.3%
Current Smoker		7582	9.4%
30-day all-cause mortality		339	0.4%
One-year all-cause mortality		2687	3.3%

Procedures *: Radiologic interventions or pain procedures under general anesthesia or monitored anesthetic care. Others **: Hinduism, Islam, and Cheondogyo. ASA, American Society of Anesthesiologists; ENT, Ear-Nose-Throat; OPH, Ophthalmic; OBGY, Obstetrics and Gynecologic; eGFR, estimated Glomerular Filtration Rate; RRT, Renal Replacement Therapy; ICU, Intensive Care Unit.

**Table 2:** Multivariate logistic regression analysis for all-cause 30-day mortality after surgery.

Variables	Odds Ratio	95% Confidence Interval		*P*-Value
		Lower Limit	Upper Limit	
Age	1.012	1.003	1.021	0.006
Educational Level				
Less than high school	1 (ref)			
More than or equal to high school, less than college	0.925	0.678	1.263	0.624
More than, equal to college	0.925	0.670	1.277	0.634
Occupation				
Office worker	1 (ref)			
Professional	1.110	0.652	1.890	0.701
Housework	0.987	0.693	1.406	0.943
Own business	1.076	0.741	1.563	0.700
Student or military	0.893	0.454	1.757	0.744
Unemployed	0.880	0.609	1.272	0.497
Religion				
None	1 (ref)			
Protestantism	0.642	0.476	0.866	0.004
Catholicism	0.855	0.565	1.292	0.456
Buddhism	0.912	0.635	1.309	0.617
Others *	55.206	0.000		0.995
Marital Status				
Never married	1 (ref)			
Married, living together	0.678	0.462	0.995	0.047
Divorced/separated	0.573	0.359	0.917	0.020
Widowed	0.709	0.351	1.429	0.336
Current Alcohol Use				
No	1 (ref)			
Yes	1.390	1.048	1.844	0.022
Quit	3.692	0.695	19.623	0.125
Past Smoking History				
Yes	1 (ref)			
Never smoked	0.872	0.608	1.251	0.458
Quit	0.252	0.047	1.341	0.106
Current Smoking (Yes)	1.344	0.882	2.047	0.168

Hosmer-Lemeshow Stats = 8.825 (*P* = 0.357). Others *: Hinduism, Islam, and Cheondogyo.

**Table 3:** Multivariate logistic regression analysis for all-cause one-year mortality after surgery.

	Odds Ratio	95% Confidence Interval		*P*-Value
		Lower Limit	Upper Limit	
Age (year)	1.001	0.998	1.004	0.570
Preoperative eGFR (mL/min/1.73 m^2^)				
>90	1 (ref)			
60–90	1.162	1.061	1.273	<0.001
30–60	1.373	1.098	1.717	0.005
<30 or RRT	1.033	0.758	1.409	0.835
ASA Class				
I	1 (ref)			
II	0.970	0.887	1.062	0.515
III, IV, V	1.153	0.940	1.415	0.171
Educational Level				
Less than high school	1 (ref)			
More than or equal to high school, Less than college	0.932	0.834	1.041	0.217
More than, equal to college	0.909	0.807	1.022	0.111
Occupation				
Office worker	1 (ref)			
Professional	1.124	0.923	1.369	0.246
Housework	1.059	0.926	1.195	0.402
Own business	1.127	0.993	1.295	0.081
Student or military	0.790	0.617	1.003	0.064
Unemployed	1.013	0.883	1.162	0.853
Religion				
None	1 (ref)			
Protestantism	0.962	0.859	1.078	0.508
Catholicism	0.948	0.819	1.097	0.473
Buddhism	0.983	0.866	1.115	0.787
Others *	2.265	0.841	6.099	0.106
Marital Status				
Never married	1 (ref)			
Married, living together	0.857	0.746	0.983	0.028
Divorced/separated	0.884	0.741	1.054	0.170
Widowed	0.883	0.692	1.126	0.316
Current Alcohol Use				
No	1 (ref)			
Yes	1.073	0.964	1.194	0.199
Quit	1.331	0.646	2.740	0.438
Past Smoking History				
Yes	1 (ref)			
Never smoked	0.954	0.834	1.092	0.495
Quit	0.739	0.356	1.533	0.417
Current Smoking: No	0.923	0.781	1.091	0.347

Hosmer-Lemeshow stat = 2.669 (*P* = 0.953). Others *: Hinduism, Islam, and Cheondogyo. eGFR, estimated Glomerular Filtration Rate; RRT, Renal Replacement Therapy; ASA, American Society of Anesthesiologists.

## Appendix A

**Table A1:** Univariate logistic regression analysis for all-cause 30-day mortality after surgery.

Variable	Odds Ratio	95% Confidence Interval		*P*-Value
		Lower Limit	Upper Limit	
Gender: female	0.980	0.790	1.215	0.851
Body mass index (kg/m^2^)	1.008	0.978	1.038	0.627
Age (year)	1.009	1.003	1.016	0.006
Type of surgery				
Cardiovascular surgery	1 (ref)			
General surgery	1.580	0.727	3.434	0.248
Neurosurgery	1.349	0.537	3.390	0.524
Spine Surgery	1.570	0.649	3.795	0.317
Thoracic Surgery	2.195	0.849	5.677	0.105
Procedures *	3.714	0.456	30.253	0.220
Orthopedic surgery	1.311	0.597	2.879	0.500
ENT, Plastic, Dental, Eye surgery	1.485	0.684	3.221	0.317
Urologic or OBGY surgery	1.394	0.640	3.039	0.403
Preoperative eGFR (mL/min/1.73 m^2^)				
>90	1 (ref)			
90–60	1.186	0.933	1.507	0.164
60–30	1.141	0.664	1.960	0.633
<30 or RRT	1.106	0.491	2.493	0.808
ASA Class				
I	1 (ref)			
II	1.231	0.987	1.534	0.065
III, IV, V	1.083	0.681	1.723	0.736
Charlson Comorbidity Index Score	0.995	0.903	1.097	0.919
Type of Anesthesia				
General anesthesia	1 (ref)			
Regional anesthesia	0.751	0.561	1.006	0.055
Monitored anesthesia care	1.018	0.766	1.352	0.903
Postoperative ICU admission	1.297	0.784	2.146	0.311
Educational level				
Less than high school	1 (ref)			
More than or equal to high school, less than college	0.876	0.663	1.158	0.354
More than, equal to college	0.805	0.607	1.069	0.135
Occupation				
Office Worker	1 (ref)			
Professional	1.040	0.617	1.755	0.882
Housework	1.065	0.781	1.452	0.691
Own business	1.104	0.780	1.562	0.578
Student or military	0.862	0.475	1.566	0.627
Unemployed	1.035	0.742	1.444	0.840
Religion: No (vs. Yes)	1.170	0.945	1.449	0.149
Protestantism	1			
Catholicism	1.315	0.873	1.983	0.190
Buddhism	1.442	0.006	2.066	0.056
Others **		0.000		0.995
None	1.393	0.077	1.801	0.051
Marriage Status				
Single	1 (ref)			
Married, living together	0.818	0.607	1.102	0.187
Divorced/separated	0.668	0.434	1.028	0.067
Widowed	1.058	0.576	1.942	0.857
Current Alcohol Use				
Yes	1 (ref)			
No	0.008	1.401	1.094	1
Quit	0.065	1.309	0.984	1
Past Smoking History				
Yes	1 (ref)			
No	1.125	0.864	1.464	0.383
Quit	1.132	0.833	1.539	0.427
Current Smoking: No	1.371	0.991	1.895	0.056

Procedures *: Radiologic interventions or pain procedures under general anesthesia or monitored anesthetic care. Others **: Hinduism, Islam, and Cheondogyo. ENT, Ear Nose and Throat; OBGY, Obstetrics and gynecologic; eGFR, estimated glomerular filtration rate; ASA, American Society of Anesthesiologists; ICU, Intensive care unit; RRT, renal replacement therapy.

## Appendix B

**Table A2:** Univariate logistic regression analysis for all-cause one-year mortality after surgery.

Variable	Odds Ratio	95% Confidence Interval		*P*-Value
		Lower Limit	Upper Limit	
Gender: female	0.983	0.910	1.063	0.669
Body mass index (kg/m^2^)	1.007	0.997	1.018	0.182
Age (year)	1.003	1.001	1.006	0.005
Type of surgery				
Cardiovascular surgery	1			
General surgery	0.905	0.640	1.278	0.569
Neurosurgery	1.142	0.761	1.713	0.522
Spine Surgery	0.942	0.647	1.373	0.758
Thoracic Surgery	0.986	0.673	1.444	0.942
Procedures *	0.876	0.506	1.519	0.638
Orthopedic surgery	0.945	0.665	1.343	0.753
ENT, Plastic, Dental, Eye surgery	0.917	0.649	1.295	0.621
Urologic or OBGY surgery	0.909	0.643	1.287	0.592
Preoperative eGFR (mL/min/1.73 m^2^)				
>90	1			
90–60	1.168	1.072	1.272	<0.001
60–30	1.424	1.149	1.765	0.001
<30 or RRT	1.126	0.839	1.512	0.429
ASA Class				
I	1			
II	1.028	0.950	1.113	0.487
III, IV, V	1.252	1.041	1.506	0.017
Charlson Comorbidity Index Score	1.019	0.982	1.056	0.317
Type of anesthesia				
General anesthesia	1			
Regional anesthesia	0.981	0.874	1.100	0.738
Monitored anesthesia care	0.984	0.890	1.086	0.743
Postoperative ICU admission	1.011	0.825	1.239	0.914
Educational level				
Less than high school	1			
More than or equal to high school, less than college	0.935	0.847	1.031	0.178
More than, equal to college	0.899	0.812	0.994	0.038
Occupation				
Office worker	1			
Professional	1.107	0.914	1.341	0.299
Housework	1.052	0.942	1.176	0.367
Own business	1.142	1.007	1.295	0.038
Student or military	0.835	0.675	1.032	0.095
Unemployed	1.075	0.953	1.213	0.239
Religion: No (vs. Yes)	1.012	0.937	1.094	0.759
Protestantism	1			
Catholicism	0.982	0.845	1.140	0.809
Buddhism	1.064	0.932	1.215	0.358
Others **	2.435	0.904	6.557	0.078
None	1.034	0.936	1.142	0.513
Marriage status				
Single	1			
Married, living together	0.972	0.878	1.075	0.576
Divorced/separated	0.979	0.832	1.153	0.803
Widowed	1.091	0.889	1.339	0.403
Current alcohol use				
Yes	1			
No	0.949	0.859	1.048	0.299
Quit	0.958	0.855	1.073	0.455
Past Smoking History				
Yes	1			
No	0.949	0.859	1.048	0.299
Quit	0.958	0.855	1.073	0.455
Current Smoking: No	0.921	0.804	1.055	0.236

Procedures *: Radiologic interventions or pain procedures under general anesthesia or monitored anesthetic care. Others **: Hinduism, Islam, and Cheondogyo. ENT, Ear Nose and Throat; OBGY, Obstetrics and gynecologic; eGFR, estimated glomerular filtration rate; ASA, American Society of Anesthesiologists; ICU, Intensive care unit; RRT, renal replacement therapy.
